# Adjunctive therapeutic effects of micronutrient supplementation in inflammatory bowel disease

**DOI:** 10.3389/fimmu.2023.1143123

**Published:** 2023-04-03

**Authors:** Yanrui Wu, Chuan Liu, Weiguo Dong

**Affiliations:** Department of Gastroenterology, Renmin Hospital of Wuhan University, Wuhan, Hubei, China

**Keywords:** inflammatory bowel disease, micronutrients, supplementation, vitamin, mineral, deficiency

## Abstract

Growing evidence suggests that micronutrient status may have some impact on the course of inflammatory bowel disease (IBD). However, micronutrient deficiencies are easily overlooked during the treatment of IBD patients. There have been many studies on micronutrient supplementation, in which several clinical trials have been conducted targeting vitamin D and iron, but the current research is still preliminary for other vitamins and minerals. This review provides an overview of the adjunctive therapeutic effects of micronutrient supplementation in IBD, to summarize the available evidence, draw the attention of clinicians to micronutrient monitoring and supplementation in patients with IBD, and also provide some perspectives for future research directions.

## 1 Introduction

Inflammatory bowel disease (IBD) is a chronic, recurrent inflammatory condition of the gastrointestinal tract, mainly including Crohn’s disease (CD) and ulcerative colitis (UC). Diarrhea, abdominal pain, bloody stools and weight loss are common clinical symptoms of IBD. As of 2017, the prevalence of IBD in many countries in North America and Europe has exceeded 0.3%, and in newly industrialized countries, the incidence of IBD is also rising, so it has become a global disease (1). However, the etiology of IBD is not yet clear. It may be that the immune system of genetically predisposed individuals is excessively activated under the action of environmental factors and gut commensals, resulting in impaired intestinal barrier function, dysbiosis and uncontrollable intestinal inflammation (2). In fact, IBD can have a huge impact on the physical, psychological and social life of patients. Some individuals suffer from long-term fatigue, depression or anxiety, and their quality of life is extremely low (3). Therefore, improving the management of patients with IBD is critical.

Currently, the treatment of IBD mainly consists of the use of aminosalicylic acid preparations, glucocorticoids, biologics and immunomodulators to control active flares and maintain long-term remission (4), but the risk of malnutrition is easily overlooked during this process, especially the lack of micronutrients. Micronutrient deficiencies are common in patients with IBD due to impaired nutrient absorption, and the fact that such patients tend to take self-imposed dietary restrictions to control symptoms (5). Micronutrients include vitamins and minerals. Vitamins that are more closely related to IBD include vitamins D, B, K, A, C, and E, and minerals include iron, zinc, and selenium. This review aims to summarize the benefits of micronutrient supplementation in animal models of IBD or clinical trials, to draw the attention of clinicians to micronutrient monitoring and supplementation in patients with IBD.

## 2 Vitamins

### 2.1 Vitamin D

In addition to the traditional role of regulating calcium and phosphorus metabolism and maintaining bone homeostasis, vitamin D is also an important immunomodulatory factor affecting intestinal homeostasis (6). The biological activity of vitamin D is mediated by the vitamin D receptor (VDR), which is expressed in normal intestinal epithelial cells and immune cells, such as macrophages, dendritic cells, B cells and T cells. Therefore, vitamin D can regulate the intestinal barrier and mucosal immunity, and also has some effect on the gut microbiota (7). Thus it can be seen that vitamin D status is closely related to IBD. Chronic diarrhea, malabsorption of nutrients, lack of sun exposure, and reduced consumption of dairy foods with vitamin D fortification are common in IBD patients, so these patients are more likely to have vitamin D deficiency than normal individuals (8). A study that included 965 IBD patients (61.9% CD and 38.1% UC) found that 29.9% had low vitamin D levels (serum 25-OH-D <30 ng/ml) (9). The definition of vitamin D deficiency is not yet uniform, and the cut-off values recommended by various guidelines are based on the effects of vitamin D on bone, with most guidelines recommending that serum 25(OH)D levels below 20–30 ng/ml (50–75 nmol/L) should be considered for vitamin D deficiency (10).

There is considerable evidence that low vitamin D status is associated with poor clinical outcomes in IBD. A systematic review including 27 studies with 8316 IBD patients (3115 UC and 5201 CD) showed that low vitamin D status was associated with increased disease activity, mucosal inflammation, poor quality of life (QOL) scores and future clinical relapse (11). Therefore, correcting vitamin D deficiency may provide some benefits to IBD patients. But it is difficult to achieve this through diet and sun exposure, and vitamin D supplementation is particularly necessary. Several clinical trials of vitamin D supplementation have been conducted in the IBD population and have shown positive effects. High-dose vitamin D supplementation can reduce the expression of proinflammatory cytokines (12), but whether it can alleviate disease activity remains controversial. There is randomized controlled trial (RCT) suggesting that vitamin D supplementation may attenuate disease activity in UC patients (13), but this effect was not observed in the clinical trial conducted by Bendix et al. (12) in 40 CD patients. Moreover, a Meta-analysis conducted by Valvano et al. (14) suggested that vitamin D supplementation can reduce the risk of clinical relapse in IBD patients, especially in CD patients in clinical remission. Supplementation with vitamin D in IBD patients has also shown some other benefits. For example, in the IBD population receiving infliximab, vitamin D supplementation may reduce the need for infliximab dose-escalation (15). Furthermore, a retrospective study in pediatrics found that low vitamin D levels in children with IBD before anti-TNF therapy may be associated with poor response to induction therapy (16). These studies suggest that vitamin D supplementation may improve responsiveness to anti-TNF therapy. Interestingly, a double-blind RCT found that oral administration of vitamin D_3_ 500 IU/d in winter and spring can prevent upper respiratory tract infections in IBD patients (17). This may be related to the immunomodulatory effects of vitamin D. In addition, vitamin D status may affect the psychological status of IBD patients, and a systematic review that included four intervention studies identified that vitamin D supplementation has a positive effect on mental health of IBD patients (18), but this result needs to be confirmed by further studies.

### 2.2 Vitamin B

There are a large variety of B vitamins, including thiamine (also known as vitamin B1), riboflavin (vitamin B2), folate (vitamin B9), and vitamin B12, among others. Folate and vitamin B12 deficiencies can lead to megaloblastic anemia, and monitoring of folate and vitamin B12 levels in IBD patients is necessary because deficiencies of these two B vitamins are common, especially in those with CD. A retrospective and comparative study that included 138 IBD patients found that 28.8% of CD patients and 8.6% of UC patients had folate deficiency, and 22.2% of CD patients and 7.5% of UC patients were vitamin B12 deficient, when deficiency of serum folate and vitamin B12 was defined as <3 ng/ml and <200 pg/ml, respectively (19). Since vitamin B12 can only be absorbed through specific receptors in the terminal ileum, CD patients who have undergone ileal resection are a high-risk group for vitamin B12 deficiency (20). Therefore, regular monitoring of folate and vitamin B12 levels in IBD patients, and timely supplementation when found to be insufficient, can prevent megaloblastic anemia. The demand for folate increases during pregnancy, so women with IBD should be supplemented with folic acid in the periconceptional period to avoid fetal neural tube defects (21). In addition, methotrexate and sulfasalazine interfere with folate synthesis or metabolism, and patients with IBD taking these two drugs should also receive appropriate folate supplementation (22). A Meta-analysis conducted by Burr et al. (23) also indicated that folate supplementation may have some preventive effect on colorectal cancer (CRC) occurring on the basis of IBD, but this conclusion needs to be demonstrated by further studies.

Thiamine may be associated with fatigue in IBD patients. A randomized controlled crossover trial conducted by Baker et al. (24) showed that oral high-dose thiamine could ameliorate chronic fatigue in patients with quiescent IBD. In this study, thiamine doses were 600-1800 mg/d based on gender and weight, and fatigue were measured by the Inflammatory Bowel Disease-Fatigue Questionnaire. Rapid depletion of thiamine in CD and UC can lead to Wernicke’s encephalopathy (WE), an acute neurological disorder (25). Oudman et al. (26) performed a case review of the emerging WE in IBD and noted that rapid treatment with high-dose thiamine (500 mg, 3 times/d) was lifesaving and relieved the core symptoms of WE.

The anti-inflammatory effects of riboflavin have been demonstrated in animal studies, such as reducing the production of proinflammatory cytokines, TNF-α, and IL-6, but it is not clear whether riboflavin alleviates inflammation directly by modulating the immune system or indirectly by altering the composition of the gut microbiome (27). A prospective clinical intervention study by von Martels et al. (28), involving 70 CD patients, showed that supplementing 100 mg riboflavin daily for 3 weeks significantly reduced serum inflammatory marker levels, increased antioxidant marker levels, and decreased disease activity index (Harvey-Bradshaw index). However, the results should also be viewed with caution, as this study was not placebo-controlled.

### 2.3 Vitamin K

No large-scale study has revealed the prevalence of vitamin K deficiency in the IBD population, and a cross-sectional study conducted in pediatric patients that enrolled 63 children with CD and 48 with UC showed that the prevalence of vitamin K deficiency was 54.0% in CD and 43.7% in UC, and was more likely to be present in CD patients with higher disease activity (29). Although studies have pointed out that vitamin K levels are negatively correlated with the clinical disease activity of CD (30), the relationship between vitamin K status and the clinical course of IBD needs to be elucidated by further studies. Vitamin K is an essential substance for the synthesis of several coagulation factors, and vitamin K deficiency will lead to abnormal coagulation function. Ohishi et al. (31) reported a case of early neonatal vitamin K deficiency bleeding (VKDB) associated with maternal CD. Therefore, in neonates whose mothers have active CD, vitamin K levels should be monitored and, if necessary, supplementation should be carried out in time to prevent VKDB in the early stage of neonates. Vitamin K is also related to bone metabolism (32). The study by Duggan et al. (33) found that the vitamin K levels of CD patients were lower than that of healthy controls, and the bone resorption rate was negatively correlated with vitamin K levels, which indicated that vitamin K deficiency may be one of the causes of osteoporosis in patients with CD. However, the RCT conducted by O’Connor et al. (34) did not observe the effect of vitamin K supplementation on improving bone health indices in adult CD patients, so larger clinical trials are needed to resolve the controversy. In addition, there is an animal study showing that vitamin K has a protective effect against dextran sulfate sodium (DSS) induced colitis, and that this effect is associated with the downregulation of IL-6 (35). But the role of vitamin K in the intestine still needs more *in vitro* and *in vivo* experiments to explore.

### 2.4 Vitamin A

Because vitamin A and its derivative retinoic acid have a wide range of effects in the body, researchers have also been curious about their role in intestinal inflammation. Previous studies have shown that vitamin A and retinoic acid play important roles in the regulation of mucosal immunity. Specifically, they affect cell integrity, cytokine production, innate immune cell activation, antigen presentation, and lymphocyte trafficking to mucosal surfaces (36). *In vitro* experiments by He et al. (37) showed that vitamin A could improve intestinal barrier function and reverse LPS-induced intestinal barrier damage by enhancing the expression of tight junction proteins. Meanwhile, *in vivo* experiments and clinical trials are also in progress with evidence that vitamin A supplementation ameliorates intestinal inflammation. In a DSS-induced UC mouse model, vitamins A supplementation by intragastric administration of 5000 IU retinyl acetate was shown to ameliorate colitis and increase the diversity of gut microbes (38). Masnadi Shirazi et al. (39) conducted a RCT in 150 UC patients with Mayo scores of 6-12, and subjects were randomly assigned to receive either 25000 IU/d vitamin A supplementation or a placebo for two months while conventional therapy (5-aminosalicylic acid) was followed throughout the trial. The results showed that the Mayo score in the vitamin A-supplemented group was significantly decreased, and the clinical remission rate and mucosal healing rate were significantly higher than those in the placebo group. However, considering the limitations such as the small sample size and short duration of this study, future studies with larger sample sizes and better designs are needed to confirm these preliminary results. Besides that, some researchers have also paid attention to β-carotene, the synthetic raw material of vitamin A. Honarbakhsh et al. (40) found that β-carotene can ameliorate dysbiosis and intestinal dysfunction in the mouse model of vitamin A deficiency.

### 2.5 Vitamins C and E

There is currently not much attention to the vitamin C status of IBD patients, but considering the reduced intake of vegetables and fruits in these patients, the risk of vitamin C deficiency is relatively increased (41). When scurvy occurs, it can lead to overt clinical symptoms (42). Therefore, screening for vitamin C levels in IBD patients with reduced intakes of vegetables and fruits is warranted. In addition, recent studies have shown that vitamin C may affect bone density, and adequate vitamin C status may have some preventive effect on osteoporosis in IBD patients (43).

A feature shared by vitamin C and vitamin E is their antioxidant effect, and it has previously been shown that vitamin E and vitamin C supplementation can reduce oxidative stress in CD patients (44). There are various forms of vitamin E, but studies on the effect of vitamin E are mostly cell experiments and animal experiments. α-tocopherol (αT) and γ-tocopherol (γT) are two forms of vitamin E, and Liu et al. (45) found that αT and γT could alleviate symptoms such as hematochezia and diarrhea in mice with DSS-induced colitis, as well as inhibit the reduction of the tight junction protein occludin. That is, vitamin E has a certain ameliorative effect on intestinal inflammation and the mucosal barrier. Furthermore, in azoxymethane (AOM) and DSS-induced murine colitis-associated colon cancer (CAC) models, vitamin E δ-tocotrienols (δTE) and its metabolites δTE-13’-carboxychromanol (δTE-13’) were shown to have some antitumor effects (46). Therefore, more preclinical studies are needed in the future to confirm the possible therapeutic role of vitamin E in IBD and IBD-associated colon tumors.

**Table 1** summarizes the aforementioned therapeutic effects of vitamin supplementation on IBD.

**Table 1:** Effects of vitamin supplementation in IBD.

Vitamins	Effects of supplementation	Literature sources	Study design	Research object
Vitamin D	Expression of proinflammatory cytokines ↓	Bendix et al, 2020 (12)	RCT	40 CD
	Risk of clinical relapse ↓	Valvano et al, 2022 (14)	Meta-analysis	–
	Need for infliximab dose-escalation ↓	Bendix et al, 2021 (15)	Observational follow-up study	28 CD were analyzed
	Preventing upper respiratory tract infections	Arihiro et al, 2019 (17)	RCT	223 IBD (168 UC and 55 CD)
	Positive effect on mental health	Głąbska et al, 2021 (18)	Systematic review	–
Folate	Preventing megaloblastic anemia	–	–	–
	Avoiding fetal neural tube defects (supplementation in the periconceptional period)	Cochrane Database, 2010 (21)	Systematic review	–
	Preventive effect on CRC occurring on the basis of IBD	Burr et al, 2017 (23)	Meta-analysis	–
Vitamin B12	Preventing megaloblastic anemia	–	–	–
Thiamine	Ameliorating chronic fatigue in patients with quiescent IBD	Bager et al, 2021 (24)	RCT	40 IBD
Riboflavin	Serum inflammatory marker levels and disease activity index ↓ Antioxidant marker levels ↑	von Martels et al, 2020 (28)	Prospective clinical intervention study	70 CD
Vitamin K	Preventing VKDB	–	–	–
Vitamin A	Mayo score ↓ Clinical remission rate and mucosal healing rate ↑	Masnadi Shirazi et al, 2018 (39)	RCT	150 UC
Vitamin C	Preventive effect on osteoporosis	Ratajczak et al, 2020 (43)	Review	–
Vitamins C and E	Oxidative stress ↓	Aghdassi et al, 2003 (44)	RCT	57 CD

RCT, Randomized controlled trial; CD: Crohn’s disease; UC: Ulcerative colitis; IBD, Inflammatory bowel disease; CRC, Colorectal cancer; VKDB, Vitamin K deficiency bleeding.

## 3 Minerals

In fact, according to the content and demand of the human body, minerals are divided into macroelements and trace elements. Macroelements include calcium, magnesium, sodium, potassium, phosphorus, chlorine and so on, while trace elements include iron, iodine, zinc, selenium, copper, manganese, chromium, cobalt, etc. Iron, zinc, and selenium are more closely related to IBD or are relatively more studied at present, all of which belong to trace elements, so this part mainly focuses on these three minerals. The therapeutic effects of iron, zinc, and selenium supplementation are listed in **Table 2**.

**Table 2:** Effects of mineral supplementation in IBD.

Minerals	Effects of supplementation	Literature sources	Study design	Research object
Iron	Iron status and hemoglobin levels ↑	–	–	–
	HRQoL score ↑	Huguet et al, 2022 (47)	Prospective, observational study	98 IBD (66 CD and 32 UC)
	Ameliorating the symptoms of RLS	Becker et al, 2018 (48)	Prospective, observational study	31 IBD (21 CD and 10 UC)
Zinc	Risk of subsequent hospitalization, surgery, and complications ↓	Siva et al, 2017 (49)	Prospective study of IBD registry	996 IBD (773 CD and 223 UC)
	Serum IL-2 and IL-10 concentrations ↓	de Moura et al, 2020 (50)	Blind interventional study	41 UC
Selenium	Cardiovascular risk associated with increased inflammatory markers ↓, especially in CD patients	Castro Aguilar-Tablada et al, 2016 (51)	Comparative study	106 IBD and 30 healthy controls

HRQoL, Health-related quality of life; RLS, Restless legs syndrome; IBD, Inflammatory bowel disease; CD, Crohn’s disease; UC, Ulcerative colitis.

### 3.1 Iron

The main reasons why patients with IBD are prone to iron deficiency are iron absorption impairment caused by chronic inflammation or intestinal resection, and iron loss caused by chronic blood loss (52). When iron deficiency occurs to a certain extent, it will affect the synthesis of hemoglobin, resulting in iron deficiency anemia (IDA). IDA is a common complication and extra intestinal manifestation of IBD patients. The prevalence of IDA was 20% in outpatients and 70% in inpatients (53), but a large number of patients were not efectively treated (54). In IBD patients, chronic fatigue that can weaken people and lead to a decline in quality of life is also closely related to anemia (55). In addition, even without progression to anemia, iron deficiency can have some overt symptoms, such as pica and restless legs syndrome (RLS) (56, 57). Therefore, strengthening the monitoring of iron levels in IBD patients and timely intervention will help improve the quality of life.

There are two forms of iron supplementation, oral iron and intravenous iron, each with its advantages and disadvantages. A series of clinical trials of iron supplementation have been carried out in IBD patients with iron deficiency. Considering that oral iron is poorly tolerated and may increase mucosal inflammation, it is only applicable to IBD patients in the quiescent phase (58). However, some new oral iron agents, such as lactoferrin and ferric maltol, have also shown good safety and tolerance, and are promising forms of iron supplements that are expected to replace standard oral iron agents (47, 59). Concerning intravenous iron, many clinicians worry about its allergic reactions (60), but more and more studies have confirmed the safety of intravenous iron supplementation (mainly ferric carboxymaltose) (61, 62), and the reaction rate of intravenous iron is higher than that of oral iron (63). In Europe, intravenous iron supplementation has been recommended as the standard treatment for iron deficiency in IBD patients (48). However, a certain proportion of patients will develop hypophosphatemia after intravenous iron, and the proportion was 26.6% in one retrospective study (64). Therefore, monitoring of serum phosphate concentrations should be strengthened during treatment.

In addition to improving iron status and hemoglobin levels, iron supplementation resulted in significant improvements in health-related quality of life (HRQoL) scores in iron-deficient IBD patients (61). Moreover, there is a clinical study demonstrating that iron supplementation in IBD patients with iron deficiency can improve the symptoms of RLS (49). Of course, iron overdose can also have side effects, but we still lack reliable data regarding the timing of stopping iron supplementation. Interestingly, an animal experiment explored intraperitoneal iron supplementation and found that it could strengthen the intestinal barrier and alleviate DSS-induced colitis in mice (65). This finding may provide a new approach to iron supplementation in IBD patients.

### 3.2 Zinc

Zinc deficiency is also common in the IBD population, and in an American cohort study of 773 patients with CD and 223 patients with UC, when serum zinc concentration <0.66 μg/ml was used as diagnostic criteria, the prevalence of zinc deficiency was 42.2% in CD and 38.6% in UC (66). The study also found that zinc deficiency was associated with an increased risk of subsequent hospitalization, surgery, as well as complications in IBD patients, and that correcting the zinc deficiency status improved these outcomes. Regarding the mechanism by which zinc deficiency adversely affects IBD, animal experiments have shown that zinc deficiency aggravates experimental colitis in mice by activating the IL-23/Th17 axis (67). Furthermore, zinc deficiency can also lead to lymphoid tissue hypoplasia, decreased natural killer (NK) cells activity, and increased apoptosis of B cells and T cells, thereby reducing the body’s immunity and increasing the risk of inflammation (68). From the current research, increasing zinc intake can reduce the risk of developing CD, as for UC, it is still controversial. In a large prospective cohort study, analysis of data from the Nurses’ Health Study with 26 years of follow-up found that zinc intake was inversely associated with the risk of CD, but supplementation with zinc, either by diet or zinc preparations, did not alter the risk of UC (69). Similar results were obtained in a French cohort study (50), in which increased dietary zinc intake was associated with a lower relative risk of CD, but no significant association with the risk of UC was observed. However, a multicenter case-control study in Japan that included 127 newly diagnosed UC patients and 171 hospital controls found that increased zinc intake had a protective effect on the development of UC by analyzing dietary zinc intake one month and one year earlier in cases and controls (70). The authors of the article also note that this is inconsistent with the results of previous cohort studies, but they consider the possible effect of ethnic differences. Clinical trials of zinc supplementation in IBD patients are still lacking, and an interventional study conducted by de Moura et al. (51) showed that zinc gluconate supplementation resulted in a significant decrease in serum IL-2 and IL-10 concentrations. Taken together, the current research evidence supports the monitoring and supplementation of zinc in IBD patients.

### 3.3 Selenium

Previous experiments have shown that selenium has effects such as attenuating oxidative stress, modulating intestinal microbiota, and blocking possible pathways involved in the progression of colitis to colon cancer (71). Serum selenium concentrations tend to be reduced in patients with IBD, especially in CD (72). The study by Barros et al. (73) found that decreased selenium levels in CD patients were associated with increased markers of oxidative stress, which further validated the role of selenium in attenuating oxidative stress. Selenium often acts in the form of selenoproteins. In addition to the above functions, selenium and antioxidant selenoproteins can regulate the differentiation of immune cells and avoid excessive immune responses (74). A multi-omics analysis revealed that selenium supplementation inhibited the onset of CD as well as the differentiation of Th1 cells through selenoprotein W (SELW)-mediated cellular reactive oxygen species scavenging (75), suggesting that selenium may have some therapeutic effects. Selenocysteine and selenocystine have also been shown in animal experiments to ameliorate DSS-induced colitis in mice by attenuating oxidative stress and intestinal inflammation (76). Furthermore, in the IBD population, adequate selenium is essential to reduce the cardiovascular risk associated with increased inflammatory markers, especially in CD patients (72). The study conducted by Short et al. (77) also found that colonic epithelial selenoprotein P (SELENOP) is the main local antioxidant in the colon. Thus, colonic SELENOP is the most effective indicator of selenium levels and activity in IBD patients and can be used to assess the risk of CAC. As for selenium supplementation, some new types of selenium supplements have emerged in recent years, such as natural selenium-enriched foods, nano-selenium, and selenium-enriched *Lactobacillus paracasei *(78–80). However, the current clinical studies on selenium supplementation in the IBD population are too preliminary, and high-quality RCTs are needed in the future to measure the effect of selenium supplementation on the course of IBD.

### 3.4 Other minerals

Sodium, potassium, and chloride among the macroelements are often not easily deficient, so less research has been conducted on these three elements. However, excessive sodium intake may have adverse effects on IBD patients. Monteleone et al. (81) found through the colitis mouse model that a high-salt diet can promote the production of inflammatory cytokines and lead to the deterioration of colitis. Some studies have also shown that high sodium intake not only promotes the conversion of naive T cells to Th1 and Th17 pro-inflammatory phenotypes, but also inhibits the function of Tregs, thus aggravating inflammation (82, 83). As for calcium, the intake of milk and dairy products is often avoided in IBD patients, especially those with symptoms of diarrhea, thus a considerable number of patients have inadequate calcium intake (84). But at present, it seems only recommended that patients treated with steroids be supplemented with calcium and vitamin D at the same time, as they are at risk of developing osteoporosis (85). Whereas the relationship between calcium intake and fracture risk in patients with IBD is still inconclusive (86, 87). The studies on magnesium are mainly animal experiments and clinical studies with small sample sizes, Trapani et al. (88) found that serum magnesium levels were negatively correlated with disease activity by analyzing serum magnesium levels, disease activity scores and C-reactive protein levels in 30 IBD patients (13 CD and 17 UC). Besides, this study also included some animal experiments. The researchers found that the expression of transient receptor potential melastatin (TRPM) 6 channel in intestinal epithelial cells was decreased in mice with DSS-induced colitis, which severely impaired the intestinal absorption of magnesium ions, but magnesium supplementation could partially restore mucosal integrity and TRPM6 expression (88). A study that included 37 IBD patients (25 UC and 12 CD) and 31 healthy people found that the hair magnesium concentration in the case group was significantly lower than that in the healthy control group. In addition, the study also focused on the relationship between magnesium levels and sleep status in patients with IBD. The authors found that hair magnesium concentration was significantly lower in patients with increased sleep latency and in those with decreased sleep duration (89). It has also been previously shown that hypomagnesemia is associated with depression in patients with IBD (90). However, whether magnesium supplementation can improve the sleep quality or psychological state of IBD patients remains to be further observed in future clinical trials.

Trace elements in addition to the above-mentioned iron, zinc, and selenium, there are iodine, copper, manganese, chromium, cobalt, etc., among which less research has been carried out on iodine, chromium, and cobalt. In general, copper deficiency may occur in IBD patients with short bowel syndrome, which is mainly manifested as sequelae of the blood system and nervous system, while the sequelae of the nervous system are often irreversible (91). Moreover, serum copper levels may be elevated in inflammatory conditions, thereby masking copper deficiency (92). Manganese deficiency is also relatively uncommon because it is present in many foods, especially plant-based foods (93). However, it has been reported that manganese levels are significantly lower in IBD patients than in healthy controls (94). Choi et al. (95) showed that in normal mice, a manganese deficient diet impaired intestinal tight junctions and thereby increased intestinal permeability. In the DSS-induced colitis mouse model, dietary manganese deficiency aggravated the degree of inflammation, whereas manganese supplementation could improve the tolerance of mice to DSS, as indicated by a milder shortening of the colon. Furthermore, Nakata et al. (96) also clarified that the missense variant A391T of the Solute Carrier Family 39 Member 8 (SLC39A8 A391T) induced impairment of intestinal mucus barrier function through down-regulation of manganese levels, thus increasing the risk of developing CD, indicating the importance of manganese for intestinal homeostasis. The above evidence suggests that patients with IBD may be at risk of manganese deficiency and that restoring manganese levels may be helpful to improve intestinal barrier function.

## 4 Discussion

The nutritional status of IBD patients tends to have some impact on the course of the disease, but the assessment of malnutrition risk and the monitoring of micronutrient levels are easily overlooked by clinicians during treatment. Clinical studies have demonstrated the benefits of micronutrient supplementation in IBD patients with specific micronutrient deficiencies. At present, micronutrients for which more clinical trials have been conducted in the IBD population are vitamin D and iron. Vitamin D supplementation in patients with IBD reduces inflammation and the risk of clinical relapse (12, 14), improves responsiveness to anti-TNF therapy (15), prevents upper respiratory tract infections in winter and spring (17), and may have a positive effect on mental health (18). However, comparisons between studies are complicated by the wide variation in dose and follow-up of existing clinical trials, and no uniform recommended supplementation dose is currently available. Vitamin D supplementation regimens should perhaps be based on body weight, to reach a certain level. Regarding iron supplementation, the main objective is to correct iron deficiency and IDA, but oral iron is less well tolerated and may increase mucosal inflammation, so patients should be considered in the quiescent or active phase of the disease when determining iron supplementation regimens (58). Although there is a risk of anaphylaxis with intravenous iron supplementation (60), many studies have confirmed its safety and efficacy, except that attention should be paid to monitoring hypophosphatemia during treatment.

IBD patients are also prone to deficiencies of other vitamins and minerals, such as vitamins B, K, A, C, and E, as well as zinc and selenium, due to impaired intestinal absorption, and restricted dietary intake (97). Supplementation of these micronutrients has also shown some benefits, for example, high-dose oral thiamine (vitamin B1) improves chronic fatigue in patients with quiescent IBD (24), vitamin A supplementation while receiving 5-aminosalicylic acid can further reduce inflammation and promote mucosal healing (39), vitamin C, vitamin E, and selenium have all been shown to reduce oxidative stress (44, 77), and correction of zinc deficiency may improve the clinical outcomes of IBD patients (66), among others. But most of these conclusions come from preliminary clinical studies, and there is still a lack of large-scale RCTs to verify them. Minerals other than iron, zinc, and selenium are relatively less prone to be deficient and therefore less well studied. In this review, we mainly discuss calcium, magnesium, and manganese. Calcium is closely related to osteoporosis in IBD patients, so it is important to pay attention to the serum calcium level in the group of patients treated with steroids (85). The research on magnesium is still in the exploratory stage, with preliminary evidence suggesting that magnesium levels may affect sleep as well as psychological status (89, 90). While a series of animal experiments have suggested that manganese is essential for the maintenance of intestinal homeostasis (95, 96).

This review summarizes the adjunctive therapeutic effects of micronutrient supplementation in IBD, but it is impractical to supplement all micronutrients in patients with IBD. Therefore, strengthening the nutritional management of IBD patients is the core, including a balanced diet, regular screening and assessment of nutritional status, and prompt supplementation when micronutrient deficiencies occur.

## Author contributions

YW performed the literature review and wrote the manuscript. CL reviewed the manuscript and provided critical comments. WD suggested the topic of the review and supervised, wrote, and critically reviewed the manuscript. All authors have read and agreed to the published version of the manuscript.
